# Effect of Melt-Mixing Duration on the Thermal Degradation and Apparent Activation Energy of Low-Loading PS/NiFe_2_O_4_ Nanocomposites: A Sensitivity-Assessed Kinetic Analysis

**DOI:** 10.3390/polym18202520

**Published:** 2026-10-15

**Authors:** Aytekin Ulutaş

**Affiliations:** Edremit School of Civil Aviation, Balıkesir University, 10300 Edremit, Balıkesir, Türkiye; aulutas@balikesir.edu.tr

**Keywords:** polystyrene, NiFe_2_O_4_, melt blending, mixing duration, thermal degradation, apparent activation energy, Kissinger method

## Abstract

PS/NiFe_2_O_4_ nanocomposites containing 2.5 wt.% NiFe_2_O_4_ were melt-blended in a twin-screw micro-compounder for 6 and 12 min (PS-NF-6 and PS-NF-12) and compared with neat PS processed for 6 min to examine whether mixing duration at a fixed low loading affects thermal degradation and apparent kinetics. XRD confirmed that the NiFe_2_O_4_ spinel structure was retained; surface SEM observations were treated as preliminary. TG data were normalized to the principal degradation step to minimize low-temperature mass-loss and balance-drift effects. PS-NF-12 degraded at higher temperatures than neat PS at all heating rates (5–20 K min^−1^), with mean shifts of 5–8 K over α = 0.05–0.80, whereas the effect of PS-NF-6 was limited to the early stage. Kissinger apparent activation energies (E_a_) were 194 ± 18, 201 ± 14, and 252 ± 12 kJ mol^−1^ for PS, PS-NF-6, and PS-NF-12, respectively, and PS-NF-12 remained the highest when any single heating rate was omitted. The KAS conversion-dependent activation energy (Eα) of PS-NF-12 increased with conversion only when the 5 K min^−1^ run was included; without it, Eα remained at 206–212 kJ mol^−1^. At constant low loading, longer mixing was thus associated with delayed mass loss and a higher peak-region E_a_.

## 1. Introduction

Polystyrene (PS) is a widely used thermoplastic polymer owing to its low cost, low density, ease of processing, and suitability for applications such as packaging, insulation, disposable products, and electrical or electronic components [1]. Nevertheless, its thermal degradation behavior remains important during processing, recycling, and applications involving elevated temperatures [1,2]. The thermal decomposition of PS proceeds through a complex radical mechanism involving random chain scission, β-scission/depolymerization, hydrogen-transfer reactions, and secondary radical reactions, with styrene monomer and oligomeric species among the major degradation products [2,3].

The kinetics of PS degradation are likewise complex. Model-free isoconversional studies have shown that the apparent activation energy of PS is not necessarily constant throughout the degradation process [3,4]. Peterson et al. reported an apparent activation energy of approximately 200 kJ mol^−1^ over most of the conversion range of PS degradation under nitrogen, whereas lower values were observed during the initial stage and were associated with degradation initiated at comparatively weak structural sites [3]. These findings demonstrate that a single global activation-energy value may not fully describe PS degradation and support the use of conversion-dependent kinetic analysis [3,4].

The incorporation of nanoscale inorganic fillers is one strategy for modifying the thermal and degradation behavior of PS. Studies on PS/clay nanocomposites have shown that organoclay contents of a few weight percent (about 3 wt.% in [5]) can alter degradation temperatures, reaction pathways, apparent activation energies, and condensed-phase behavior [5,6,7,8]. Bourbigot et al. reported that, under nitrogen, the onset of degradation of a PS–montmorillonite nanocomposite containing about 3 wt.% organo-modified clay was about 50 K higher than that of neat PS, whereas in air the onset temperature was not increased, and that the degradation kinetics of the nanocomposite differed from those of neat PS [5]. Jang and Wilkie further demonstrated that clay incorporation modified the PS degradation pathway by increasing the contribution of inter-chain radical-transfer and recombination reactions relative to neat PS [6].

Isoconversional studies provide additional evidence that the influence of a nanofiller may vary throughout degradation. Vyazovkin et al. demonstrated that introduction of a clay phase into PS increased the apparent activation energy and altered its dependence on conversion [7,8]. Such behavior is especially relevant to polymer nanocomposites because the measured thermal response may reflect several simultaneous contributions, including filler distribution, polymer–particle interfacial effects, mass transport of degradation products, and changes in the relative importance of degradation reactions as conversion proceeds [5,6,7,8]. Accordingly, kinetic evaluation at fixed conversion levels can provide information that is not captured by peak-temperature methods alone [4].

Among inorganic nanofillers, spinel ferrites are attractive for polymer-based composites because they combine chemical stability with useful magnetic and electrical characteristics. Nickel ferrite (NiFe_2_O_4_) is a cubic inverse-spinel ferrite and is known for comparatively high electrical resistivity, chemical stability, and ferrimagnetic behavior; its measured structural and magnetic properties depend on particle size, cation distribution, defects, dimensionality, and synthesis history [9]. These features have promoted the use of NiFe_2_O_4_ in magnetic, electromagnetic, sensing, and composite applications [9].

NiFe_2_O_4_ has previously been incorporated into PS. Zhao et al. prepared NiFe_2_O_4_–PS composites by hot pressing and investigated their microwave-absorbing characteristics using ferrite contents of 35, 45, 55, and 65 wt.% [10]. That study focused on complex permittivity, complex permeability, and microwave reflection loss rather than on the thermal degradation kinetics of the polymer matrix [10]. Thus, although NiFe_2_O_4_/PS composites are not new as a material combination, the experimental conditions and scientific objective of that work differ from low-loading melt-blended systems designed to examine thermal degradation behavior.

Processing history is another important variable in polymer nanocomposites because nanoparticle dispersion evolves during mixing and is influenced by polymer viscosity, particle–particle interactions, shear history, mixing geometry, and residence or mixing time [11,12]. Rishi et al. directly demonstrated that mixing time affects nanoscale dispersion in filled polymer systems and described dispersion as a kinetic process governed by both filler characteristics and the polymer environment [11]. More generally, recent reviews of polymer-composite compounding emphasize the need to balance adequate filler distribution and dispersion against excessive thermal and shear history [12].

The influence of mixing duration on thermal degradation has also been demonstrated experimentally in melt-processed metal-oxide/polymer nanocomposites. Ulutaş reported that changing the mixing duration from 6 to 12 min in NiO/PMMA nanocomposites altered nanoparticle agglomeration, thermal stability, and apparent activation energies, demonstrating that prolonged mixing does not necessarily lead to improved thermal behavior [13]. In a more directly comparable NiFe_2_O_4_-containing system, controlled melt mixing of PMMA/NiFe_2_O_4_ nanocomposites showed that mixing duration produced clear differences in nanoparticle dispersion and degradation kinetics even at constant filler content and otherwise fixed processing conditions [14]. In a companion study using the same PS batch and processing protocol, PS/Co_3_O_4_ nanocomposites mixed for 12 min showed the highest peak-based apparent activation energy, whereas isoconversional analysis did not preserve the same ordering [15]. These results indicate that the relationship between mixing duration, morphology, and thermal degradation is not necessarily monotonic and may depend on both the polymer matrix and the filler [13,14].

A matrix dependence of this kind is plausible because PMMA and PS degrade by different routes. PMMA degrades predominantly by depolymerization initiated at chain ends and weak links, yielding mainly monomer [16], whereas PS degrades by random chain scission, β-scission, and intermolecular hydrogen transfer, yielding monomer together with dimers, trimers, and other oligomers [2,3,6]. A filler whose influence depends on local confinement of chain ends may therefore affect the two matrices differently from a filler whose influence depends on the total polymer–particle contact area or on the transport of volatile products. Comparing the response of PS/NiFe_2_O_4_ with that of PMMA/NiFe_2_O_4_ prepared with the same protocol [14] and with that of PS/Co_3_O_4_ thus provides a first test of whether the effect of mixing duration is governed mainly by the matrix or by the filler.

Despite these developments, the available literature leaves an important gap concerning PS/NiFe_2_O_4_ nanocomposites. Previous work on NiFe_2_O_4_/PS has mainly emphasized electromagnetic functionality at comparatively high ferrite contents [10], whereas the combined effects of low NiFe_2_O_4_ loading and melt-mixing duration on the non-isothermal and conversion-dependent thermal degradation kinetics of PS remain insufficiently investigated. In particular, limited information is available on how mixing duration affects early-stage mass loss, the maximum degradation-rate region, and the evolution of apparent activation energy with conversion in low-loading PS/NiFe_2_O_4_ nanocomposites. Moreover, kinetic parameters of polymer nanocomposites are frequently derived from single measurements at a few heating rates without assessing how much they depend on individual data points, although differences of a few kelvin in peak temperature can translate into large differences in apparent activation energy.

Accordingly, the present study investigates PS/NiFe_2_O_4_ nanocomposites containing 2.5 wt.% NiFe_2_O_4_ prepared by twin-screw melt blending using mixing durations of 6 and 12 min under otherwise identical processing conditions. X-ray diffraction (XRD) was used to evaluate the structure of the filler after processing, surface scanning electron microscopy (SEM) provided preliminary morphological observations, and differential thermal analysis (DTA) and thermogravimetric analysis (TG/DTG) at four heating rates were used to characterize the degradation response. Peak-based apparent activation energies were evaluated using the Kissinger and Takhor methods, whereas the Kissinger–Akahira–Sunose (KAS) isoconversional method was applied to determine the evolution of apparent activation energy with conversion. The uncertainty of all kinetic parameters was assessed by regression statistics, a leave-one-heating-rate-out test, and a Monte Carlo perturbation of the peak temperatures. The novelty of this work does not lie in the PS/NiFe_2_O_4_ material combination, which has been reported previously [10], but in two aspects: (i) the comparison of two melt-mixing durations at a fixed low filler loading within a series that covers two matrices and two oxide fillers processed with the same protocol, and (ii) a sensitivity assessment that identifies which of the resulting kinetic differences are robust when replicate measurements are not available.

## 2. Materials and Methods

### 2.1. Materials

Polystyrene (PS; weight-average molecular weight, Mw ≈ 35,000 g mol^−1^; CAS No. 9003-53-6) was purchased from Acros Organics (Geel, Belgium) in powder form. Nickel ferrite (NiFe_2_O_4_) nanopowder (average particle size < 50 nm; CAS No. 12168-54-6; Sigma-Aldrich, Merck KGaA, Darmstadt, Germany) was used as the inorganic filler. Both materials were used as received, without chemical modification or compatibilization.

### 2.2. Preparation of Neat PS and PS/NiFe_2_O_4_ Nanocomposites

Melt blending was carried out in a twin-screw micro-compounder (DSM Xplore, Sittard, The Netherlands; screw diameter 5 mm, L/D = 20) with a chamber capacity of 4 g. For each nanocomposite batch, 3.9 g of PS and 0.1 g of NiFe_2_O_4_ were dry-premixed before feeding, giving a filler content of 2.5 wt.% relative to the total composite mass. The three barrel-zone temperatures were set to 473, 483, and 483 K, and the screw speed was fixed at 100 rpm. Two mixing durations, 6 and 12 min, were applied to the nanocomposites while all other processing parameters were kept unchanged; the resulting samples are designated PS-NF-6 and PS-NF-12. Neat PS was processed under the same conditions for 6 min and is designated PS. A neat PS control processed for 12 min was not prepared; the consequences of this limitation are discussed in Section 3.7. The two mixing durations were selected for comparison and were not intended as a formal optimization of mixing time.

**Figure 1:**
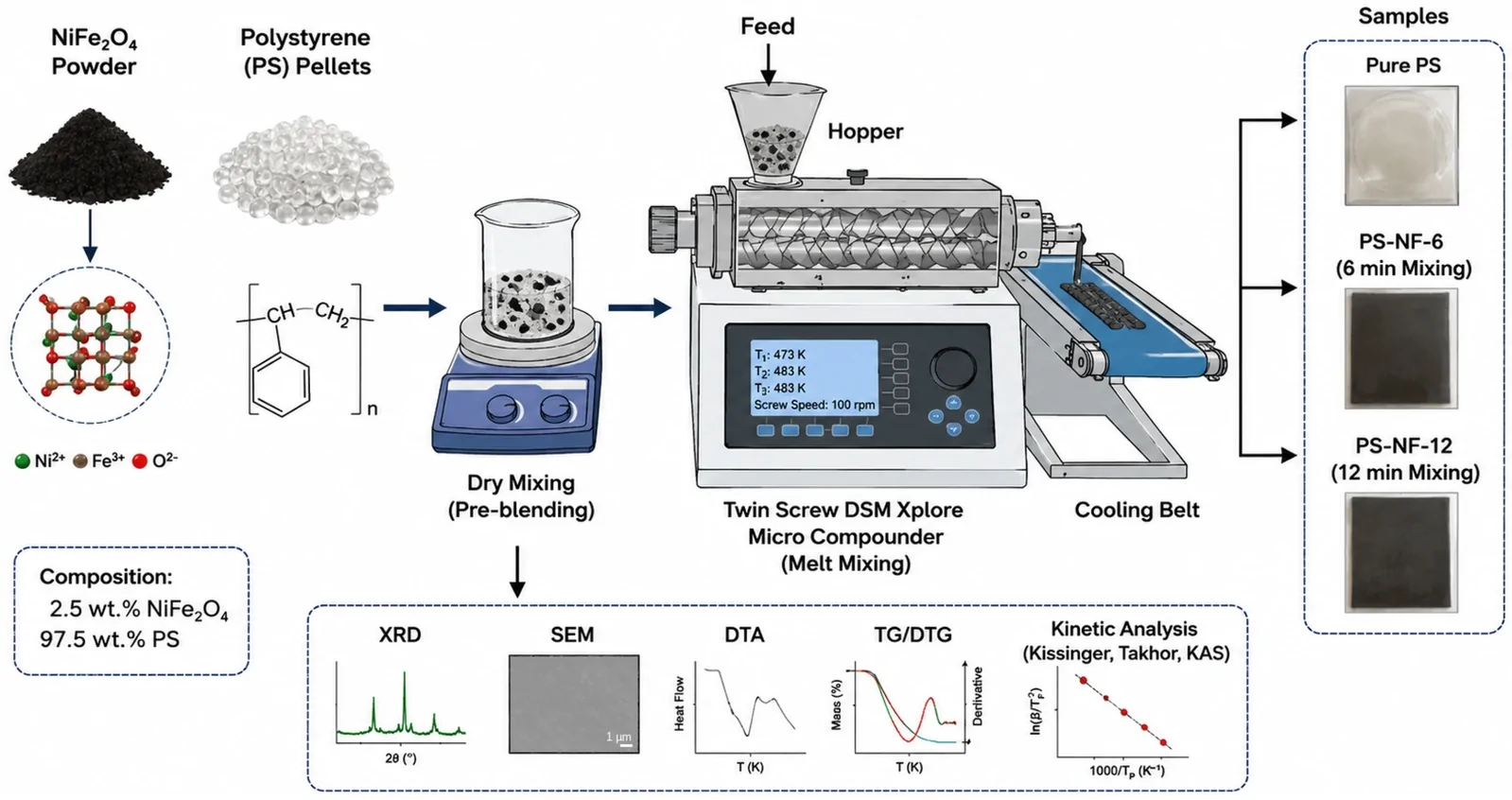
Schematic illustration of the preparation of neat PS and PS/NiFe_2_O_4_ nanocomposites by melt blending. The SEM inset shows the neat PS sample; the other characterization plots are schematic representations.

A schematic representation of the overall experimental route, including the preparation of neat PS and PS/NiFe_2_O_4_ nanocomposites with different mixing durations by melt blending, is presented in Figure 1.

### 2.3. X-Ray Diffraction

**Figure 2:**
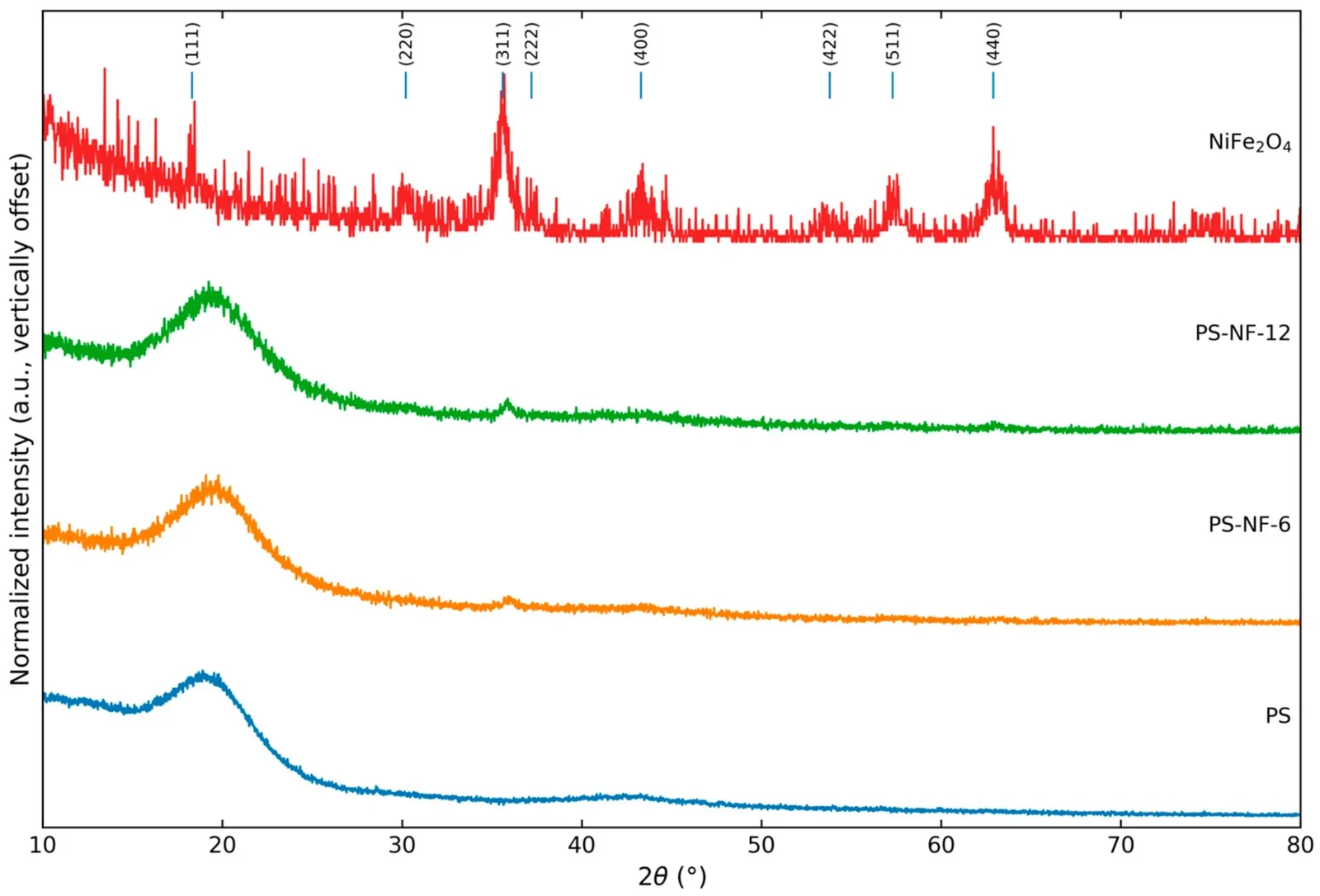
XRD patterns of NiFe_2_O_4_ nanopowder, neat PS, PS-NF-6, and PS-NF-12.

Phase identification and structural characterization were performed by X-ray diffraction (XRD) using a Bruker D8 Advance diffractometer (Bruker AXS GmbH, Karlsruhe, Germany) with CuKα radiation (λ = 0.154056 nm). Diffraction patterns were recorded over a 2θ range of 5–80° with a step size of 0.013°; the 10–80° region is presented in Figure 2. The patterns of neat PS, NiFe_2_O_4_ nanopowder, PS-NF-6, and PS-NF-12 were compared to evaluate the amorphous contribution of the polymer matrix, the characteristic reflections of the spinel ferrite phase, and any detectable processing-related crystallographic changes. Reflections were indexed to cubic spinel NiFe_2_O_4_ (space group Fd-3m) according to the reference pattern of NiFe_2_O_4_ [17].

### 2.4. Scanning Electron Microscopy

The outer surface of the extruded specimens was examined by scanning electron microscopy (SEM) using a JEOL JSM-6060LV microscope (JEOL Ltd., Tokyo, Japan) operated at an accelerating voltage of 10 kV (5 kV for neat PS). Before observation, the specimens were sputter-coated with a thin gold layer to minimize charging. For direct comparison among the three formulations, representative micrographs recorded at the same magnification (×5000) were used in the present study. Because the SEM observations were limited to the specimen surface and no elemental mapping was performed, they are treated as preliminary, qualitative observations and were not used to quantify bulk filler dispersion or to support mechanistic conclusions.

### 2.5. Thermal Analysis

Differential thermal analysis (DTA) and thermogravimetric analysis (TG/DTG) were performed using a Hitachi Exstar SII 7300 simultaneous thermal analysis system (Hitachi High-Tech, Tokyo, Japan). Samples of 10.0–16.4 mg (the initial mass of each specimen is listed in Table 1) were placed in alumina crucibles, with an empty alumina crucible as reference, and heated from 303 to 1073 K under nitrogen (99.99%) flowing at 50 mL min^−1^. Data were recorded every 0.5 s. Temperatures were measured with the instrument’s built-in thermocouple using the manufacturer’s default calibration settings; no additional user-defined temperature correction was applied. Non-isothermal measurements were carried out at heating rates (β) of 5, 10, 15, and 20 K min^−1^. The principal endothermic DTA peak temperature (T_x_) and the temperature of the maximum mass-loss rate (T_x_,DTG) were determined for each heating rate as the temperatures of the respective extrema within the main degradation region. DTG curves were used as exported by the instrument software, without smoothing, baseline, or slope corrections; recalculating T_x_,DTG from Savitzky–Golay-smoothed curves (window ≈ 3 K) changed the Kissinger activation energies by at most 5 kJ mol^−1^. One measurement was performed for each sample and heating rate; replicate measurements were not available, and the uncertainty of the derived parameters was therefore assessed numerically (Section 2.6). The neat PS measurements were shared with a companion study on PS/Co_3_O_4_ nanocomposites that used the same PS batch and identical processing and thermal-analysis conditions [15].

### 2.6. Data Treatment and Kinetic Analysis

All neat PS curves showed a gradual mass loss of approximately 2.2–2.5% below 573 K and a negative apparent residue at high temperature, indicating balance drift that could not be removed by blank subtraction because no blank curves were recorded. To minimize these effects, all TG curves were normalized to the principal degradation step:

(1) α = (m_573_ − m_T_)/(m_573_ − m_773_)

where m_573_ and m_773_ are the masses at 573 and 773 K, respectively, and m_T is the mass at temperature T. The characteristic temperatures T_5_, T_10_, T_30_, T_50_, and T_80_ were defined as the temperatures at α = 0.05, 0.10, 0.30, 0.50, and 0.80, respectively, and were determined at all four heating rates. Because of this normalization, the neat PS values differ from those reported in [15].

The apparent activation energy associated with the dominant degradation-rate region was first evaluated using the Kissinger and Takhor peak-based methods. For both approaches, the T_x_,DTG values at 5, 10, 15, and 20 K min^−1^ were used as the primary data set, and the DTA peak temperatures (T_x_) were used as an independent check. The Kissinger relationship was expressed as

(2) ln(β/T_x_^2^) = −E_a_/(RT_x_) + C

where β is the heating rate, T_x_ is the absolute peak temperature (T_x_,DTG for the primary data set, and the DTA peak temperature for the independent check), E_a_ is the apparent activation energy, R is the universal gas constant, and C is a constant. The Takhor relationship was written as

(3) ln(β) = − E_a_/(RT_x_) + C

The activation energy was obtained from the slope of the corresponding linear regression for each sample. Equation (3) does not include the Doyle approximation factor (1.052) used in the Ozawa–Flynn–Wall method; the Takhor values are therefore systematically higher than the Kissinger values, and the method is retained for comparability with the companion study.

To evaluate the conversion dependence of the degradation barrier, the model-free Kissinger–Akahira–Sunose (KAS) isoconversional method was additionally applied to the TG data. The extent of conversion was calculated according to Equation (1). Temperatures corresponding to fixed conversion levels from α = 0.10 to 0.80 were determined at each heating rate, and the apparent activation energy at each conversion level was calculated from

(4) ln(β/Tα^2^) = −Eα/(RTα) + Cα

where Tα is the absolute temperature corresponding to a fixed conversion level and Eα is the conversion-dependent apparent activation energy. The peak-based and isoconversional approaches were therefore treated as complementary: the former characterizes the vicinity of the maximum degradation rate, whereas the latter evaluates changes in the apparent kinetic barrier as degradation progresses.

Because replicate measurements were not available, the uncertainty of E_a_ and Eα was assessed in three ways: (i) the standard error of the regression slope (*n* = 4), reported as ± values; (ii) a leave-one-heating-rate-out test, in which the analysis was repeated four times, omitting each heating rate in turn; and (iii) a Monte Carlo perturbation in which normally distributed errors (σ = 1 K) were added to the peak temperatures (20,000 iterations) to obtain 95% intervals. A result was considered robust only if it was preserved in all leave-one-out subsets.

## 3. Results and Discussion

### 3.1. XRD Characterization

The X-ray diffraction (XRD) patterns of neat PS, NiFe_2_O_4_ nanopowder, and the PS/NiFe_2_O_4_ nanocomposites prepared at mixing durations of 6 and 12 min are presented in Figure 2. Neat PS is dominated by a broad diffuse halo, confirming the predominantly amorphous nature of the polymer matrix. In contrast, the NiFe_2_O_4_ nanopowder exhibits distinct crystalline reflections characteristic of cubic spinel nickel ferrite. The principal reflections expected for NiFe_2_O_4_ occur at approximately 2θ = 18.3°, 30.2°, 35.6°, 37.2°, 43.3°, 53.8°, 57.3°, and 62.9°, corresponding to the (111), (220), (311), (222), (400), (422), (511), and (440) crystallographic planes, respectively (JCPDS card No. 10-0325) [17]. These positions agree closely with the diffraction pattern obtained for the NiFe_2_O_4_ powder used in the present study. The intense reflection near 35.6° is associated with the characteristic (311) plane of the cubic spinel structure.

Following incorporation of 2.5 wt.% NiFe_2_O_4_ into PS, the diffraction profiles of PS-NF-6 and PS-NF-12 remain dominated by the broad amorphous contribution of the polymer matrix. Nevertheless, a weak reflection corresponding to the principal (311) peak of NiFe_2_O_4_ can still be distinguished, whereas the higher-angle (440) reflection is only marginally discernible. The comparatively low intensity of these filler-related reflections is consistent with the low inorganic loading and the dominant scattering contribution of the amorphous PS matrix. A similar coexistence of an amorphous polymer contribution with retained crystalline NiFe_2_O_4_ reflections has been observed in melt-processed polymer/NiFe_2_O_4_ systems [14].

Importantly, the characteristic NiFe_2_O_4_ reflections remain within essentially the same diffraction regions in both PS-NF-6 and PS-NF-12. No additional well-defined reflections attributable to a new crystalline phase appear after increasing the melt-mixing duration from 6 to 12 min. Within the resolution of the present XRD measurements, these observations indicate that the fundamental spinel structure of NiFe_2_O_4_ was retained during melt processing and that extending the mixing duration did not induce a detectable crystallographic phase transformation.

### 3.2. Morphology Characterization

**Figure 3:**
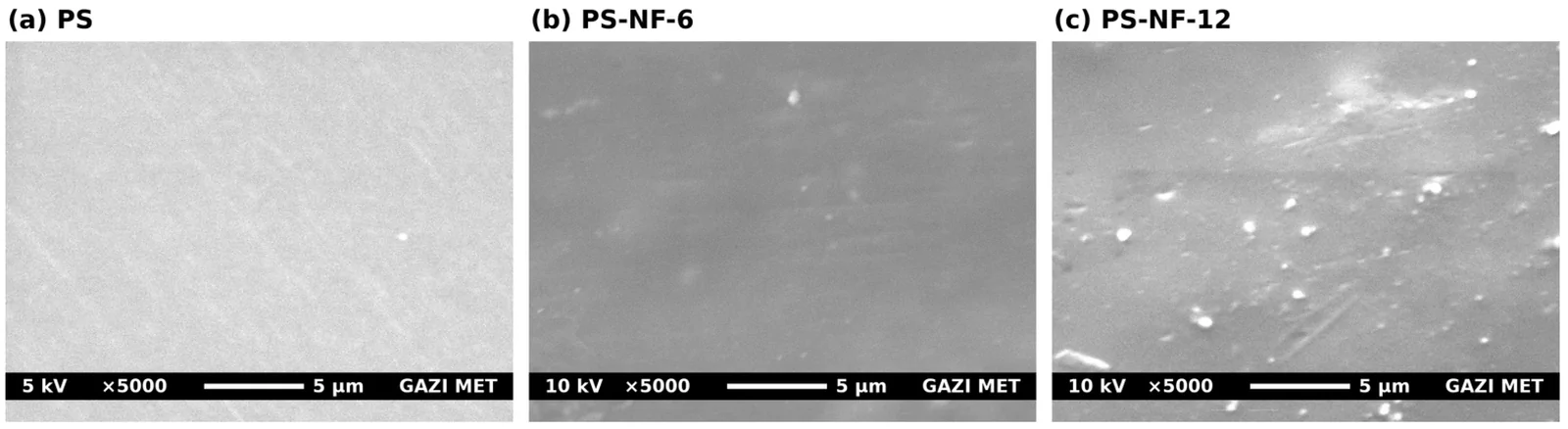
Representative SEM micrographs of (**a**) neat PS, (**b**) PS-NF-6, and (**c**) PS-NF-12 obtained at ×5000 magnification (accelerating voltage: 5 kV for neat PS and 10 kV for the nanocomposites).

Representative SEM micrographs of neat PS, PS-NF-6, and PS-NF-12 obtained at the same magnification are presented in Figure 3. The surface of neat PS is comparatively smooth and featureless, as expected for a polymer sample without an intentionally incorporated inorganic dispersed phase; the lighter overall contrast of Figure 3a partly reflects the lower accelerating voltage used for this sample. After addition of 2.5 wt.% NiFe_2_O_4_, the near-surface morphology changes visibly.

PS-NF-6 retains a relatively smooth polymer-rich background, although isolated brighter contrast features are distributed over the examined region. Considering the formulation and the XRD confirmation of the crystalline ferrite phase, these features are consistent with filler-containing or filler-rich near-surface regions. Nevertheless, SEM contrast alone cannot provide unequivocal compositional identification, because bright features in secondary-electron images may also arise from surface topography, coating non-uniformity, or charging; they are therefore interpreted qualitatively rather than assigned directly to individual NiFe_2_O_4_ particles.

A more heterogeneous appearance is observed for PS-NF-12. The number of visible contrast features increases and localized regions of greater apparent size are also present. These differences between single representative fields suggest, but do not establish, that the near-surface morphology differs between the two processing conditions. Such processing sensitivity would be consistent with the established role of mixing time in the kinetic evolution of nanofiller dispersion. Rishi et al. showed that nanoscale filler dispersion depends on mixing duration and the polymer-filler environment rather than simply progressing toward an indefinitely finer state with increasing processing time [11]. The simultaneous presence of numerous small contrast features and localized larger regions in PS-NF-12 is therefore not interpreted as evidence of improved dispersion. In PMMA/NiFe_2_O_4_ nanocomposites prepared with the same protocol, the 6 and 12 min conditions also produced different morphologies [14].

The present SEM observations provide a qualitative comparison of representative near-surface regions and should not be regarded as a quantitative measure of three-dimensional filler dispersion throughout the bulk composite. Quantitative particle/agglomerate size distributions or direct chemical identification of the observed contrast features would require complementary cross-sectional imaging and compositional analysis such as EDS mapping. In addition, only one field per formulation is presented. The morphological observations are therefore not used to interpret the thermal and kinetic results in the following sections.

### 3.3. Thermogravimetric Analysis

**Figure 4:**
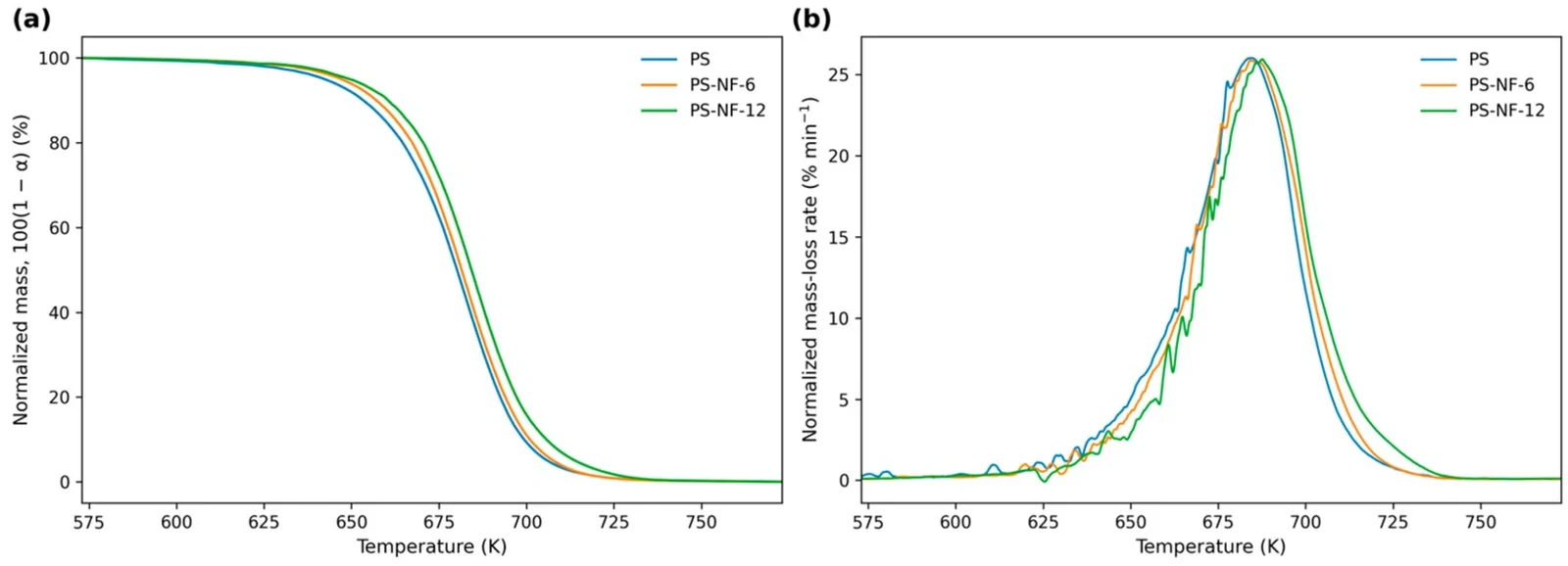
(**a**) Normalized TG and (**b**) normalized DTG curves of neat PS, PS-NF-6, and PS-NF-12 at 10 K min^−1^. The curves are normalized to the principal degradation step (573–773 K) according to Equation (1).

The TG and DTG curves of neat PS, PS-NF-6, and PS-NF-12 at 10 K min^−1^, normalized to the principal degradation step according to Equation (1), are shown in Figure 4. All samples exhibit a single dominant mass-loss step, indicating that degradation remains governed by decomposition of the PS matrix, which involves random chain scission, depolymerization, and secondary radical reactions [1,2,3]. Without this normalization, the low-temperature mass loss of neat PS (Section 2.6) would artificially lower its early-stage characteristic temperatures. The characteristic temperatures at all four heating rates, together with the DTG and DTA peak temperatures and the initial specimen masses, are listed in Table 1.

At 10 K min^−1^, T_5_ increases from 642.3 K for neat PS to 647.1 K for PS-NF-6 and 649.6 K for PS-NF-12, corresponding to shifts of 4.8 and 7.3 K, respectively. PS-NF-12 also shows higher T_50_ (684.3 K) and T_80_ (697.2 K) values than neat PS (680.2 and 692.5 K) and PS-NF-6 (681.5 and 694.0 K). In contrast, the DTG peak temperatures differ only slightly (684.6, 684.2, and 687.6 K, respectively).

Because replicate measurements are not available, the consistency of these shifts across the four independently measured heating rates provides the main evidence for their reproducibility. PS-NF-12 degrades at a higher temperature than neat PS at every heating rate and every characteristic conversion in Table 1; the smallest shift is 2.5 K and the mean shifts range from 5.2 to 7.9 K. PS-NF-12 is also higher than PS-NF-6 in all twenty comparisons (smallest shift 1.0 K). The effect of PS-NF-6 is confined to the early stage: T_5_ and T_10_ exceed those of neat PS by 3.9 and 3.1 K on average, whereas from T_30_ onward the differences are within −2.3 to +1.9 K and change sign between heating rates. The higher characteristic temperatures of PS-NF-12 cannot be attributed to thermal lag, since its 5 K min^−1^ specimen was among the lightest (10.4 mg) yet showed the highest characteristic temperatures at that heating rate.

The magnitude of these shifts is modest, and without replicate measurements their absolute values should be regarded as indicative. Non-monotonic effects of inorganic fillers on the degradation kinetics of PS have also been reported for PS/CdS composites [18]. The present data do not establish whether the higher degradation temperatures of PS-NF-12 originate from polymer–filler interactions, transport of volatile products, filler organization, or the additional thermo-mechanical history of the matrix; these possibilities are discussed in Section 3.7.

### 3.4. Differential Thermal Analysis

**Figure 5:**
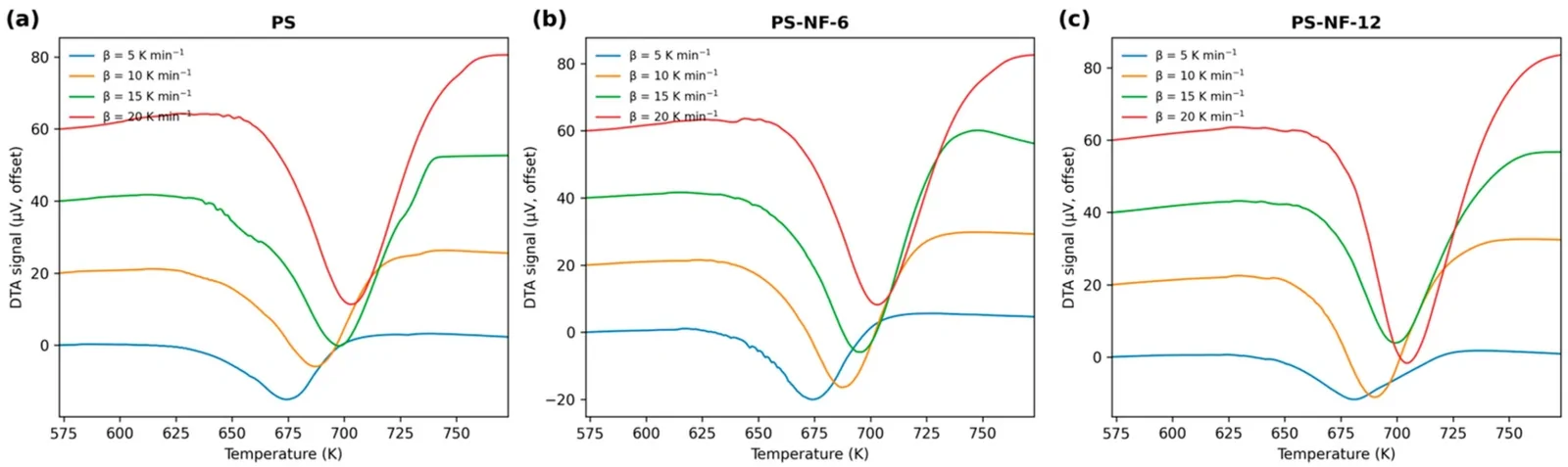
DTA curves of (**a**) neat PS, (**b**) PS-NF-6, and (**c**) PS-NF-12 at heating rates of 5, 10, 15, and 20 K min^−1^.

The DTA curves of neat PS, PS-NF-6, and PS-NF-12 recorded at heating rates of 5, 10, 15, and 20 K min^−1^ are shown in Figure 5. All samples exhibit a dominant endothermic event associated with the main thermal degradation region of the PS matrix. Incorporation of 2.5 wt.% NiFe_2_O_4_ does not introduce an additional dominant thermal event within this region, indicating that the overall high-temperature DTA response remains principally governed by degradation of PS. For all samples, the characteristic degradation peak shifts toward higher temperature as the heating rate increases. Such behavior is characteristic of non-isothermal polymer degradation and is consistent with the established heating-rate dependence of PS degradation kinetics [3,4].

The DTA degradation-peak temperatures obtained at different heating rates are listed in Table 1. For neat PS, T_x_ increases from 674.5 K at 5 K min^−1^ to 703.0 K at 20 K min^−1^. PS-NF-6 displays closely comparable values of 674.3, 687.0, 695.5, and 703.1 K at 5, 10, 15, and 20 K min^−1^, respectively, indicating that the 6 min processing condition produces only a limited change in the main DTA degradation-peak position.

A more distinct response is observed for PS-NF-12, for which T_x_ values of 681.3, 690.1, 699.3, and 704.4 K are obtained at heating rates of 5, 10, 15, and 20 K min^−1^, respectively. The peak is consistently shifted to higher temperature relative to neat PS, although the magnitude of the difference decreases as the heating rate increases. The largest displacement occurs at 5 K min^−1^, where PS-NF-12 is approximately 6.8 K higher than neat PS, whereas the difference is only about 1.4 K at 20 K min^−1^. Thus, the difference associated with the longer mixing duration is largest at the lowest heating rate and decreases as the heating rate increases. These observations support the use of conversion-dependent kinetic analysis rather than relying on a single degradation-peak temperature.

### 3.5. Kinetic Analysis of Thermal Degradation

The thermal degradation kinetics were evaluated using complementary peak-based and isoconversional approaches. Kissinger and Takhor analyses were applied to the maximum degradation-rate temperatures obtained from DTG curves, while the KAS method was used to determine the variation in apparent activation energy with conversion. This distinction is important because peak-based methods mainly characterize the vicinity of the maximum reaction rate, whereas isoconversional approaches evaluate whether the apparent kinetic barrier varies throughout the degradation process. ICTAC recommendations emphasize the value of multiple temperature programs and isoconversional analysis when kinetic complexity is suspected [4].

**Figure 6:**
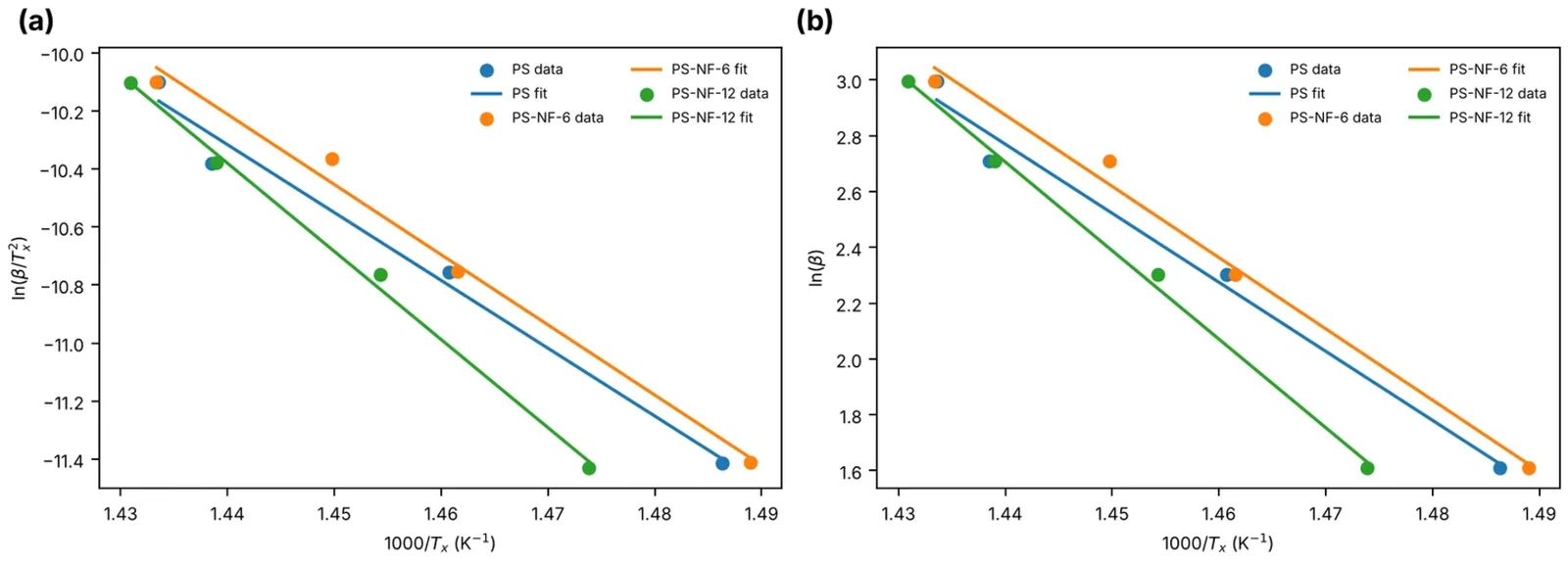
Peak-based kinetic analysis of neat PS and PS/NiFe_2_O_4_ nanocomposites: (**a**) Kissinger and (**b**) Takhor plots constructed from DTG maximum degradation-rate temperatures.

The Kissinger and Takhor plots constructed from the DTG peak temperatures are presented in Figure 6a,b, respectively. The corresponding apparent activation energies, their standard errors, and the Kissinger values obtained from the DTA peak temperatures are summarized in Table 2; the peak temperatures are listed in Table 1. The Kissinger method yields apparent activation energies of 194.3 ± 18.1, 201.1 ± 14.4, and 252.4 ± 11.5 kJ mol^−1^ for neat PS, PS-NF-6, and PS-NF-12, respectively. The Takhor method gives values that are higher by about 11 kJ mol^−1^ (205.7, 212.4, and 263.8 kJ mol^−1^), consistent with the absence of the Doyle factor in Equation (3). Both approaches give the same ordering of the values, PS < PS-NF-6 < PS-NF-12. The neat-PS Kissinger value of 194.3 kJ mol^−1^ is consistent with the magnitude reported by Peterson et al. for the principal thermal-degradation region of PS under nitrogen, where apparent activation energies close to 200 kJ mol^−1^ were obtained [3]. This agreement provides an independent reference point for the present kinetic calculations. The difference between neat PS and PS-NF-6 (6.8 kJ mol^−1^) is smaller than the standard error of either value; so, the two samples cannot be distinguished. The difference between PS-NF-12 and neat PS (58.1 kJ mol^−1^) is about 2.7 times their combined standard error (21.4 kJ mol^−1^).

With only four heating rates, a high coefficient of determination does not by itself imply a small uncertainty in E_a_; for neat PS, for example, the 95% confidence interval of the Kissinger slope corresponds to about ±78 kJ mol^−1^. The robustness of the ordering was therefore tested as described in Section 2.6, and the results are given in Table 3. When any single heating rate was omitted, the Kissinger value of PS-NF-12 (231.0–256.2 kJ mol^−1^) remained higher than those of neat PS (180.5–207.1 kJ mol^−1^) and PS-NF-6 (189.6–217.5 kJ mol^−1^) in every subset. Perturbing the peak temperatures with σ = 1 K gave 95% intervals of 174–215, 180–225, and 221–289 kJ mol^−1^, respectively; the interval of PS-NF-12 does not overlap that of neat PS and overlaps only marginally with that of PS-NF-6, whereas the intervals of neat PS and PS-NF-6 largely coincide. The DTA peak temperatures, obtained from an independent signal, give the same ordering (176.1 ± 8.7, 180.0 ± 8.6, and 221.4 ± 20.2 kJ mol^−1^). Taken together, the higher peak-region apparent activation energy of PS-NF-12 is supported by all tests, whereas neat PS and PS-NF-6 are indistinguishable within the present uncertainty. These values are apparent parameters of the maximum-rate region rather than kinetic constants of the complete mass-loss process [3,7,8], and the KAS analysis in Section 3.6 examines whether they vary with conversion.

### 3.6. KAS Isoconversional Analysis

**Figure 7:**
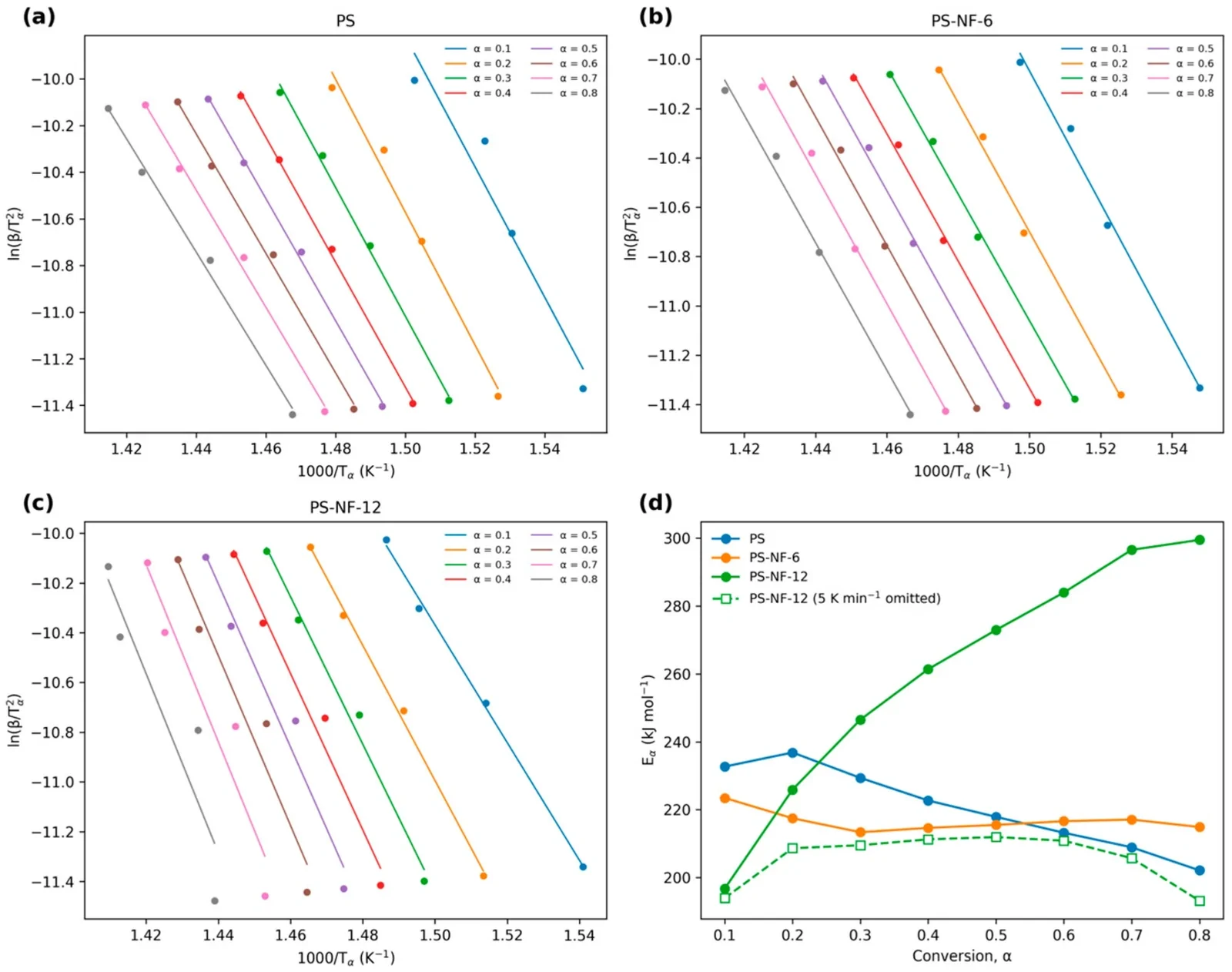
KAS isoconversional analysis: KAS plots for (**a**) neat PS, (**b**) PS-NF-6, and (**c**) PS-NF-12, and (**d**) apparent activation energy (Eα) as a function of conversion (α). In (**a**–**c**), symbols are the experimental data obtained at 5, 10, 15, and 20 K min^−1^, lines are linear fits, and colors indicate the conversion level (α) given in the legend. The dashed line shows PS-NF-12 with the 5 K min^−1^ measurement omitted.

The KAS isoconversional plots for neat PS, PS-NF-6, and PS-NF-12, together with the variation in apparent activation energy as a function of conversion, are presented in Figure 7a–d. The corresponding apparent activation energies and coefficients of determination at each conversion level are summarized in Table 4. The KAS analysis was performed at conversion levels from α = 0.10 to 0.80 using TG data acquired at all four heating rates. Neat PS shows a generally decreasing apparent activation-energy profile after the initial conversion region: Eα is 232.7 kJ mol^−1^ at α = 0.10, reaches 236.8 kJ mol^−1^ at α = 0.20, and subsequently decreases to 202.1 kJ mol^−1^ at α = 0.80. Conversion-dependent activation energies for PS and PS-based nanocomposites have previously been reported using isoconversional analysis, supporting the conclusion that degradation cannot always be represented adequately by a single constant kinetic barrier [3,7,8]. PS-NF-6 shows a considerably narrower Eα interval; after decreasing from 223.5 kJ mol^−1^ at α = 0.10 to 213.4 kJ mol^−1^ at α = 0.30, Eα remains approximately within 214–217 kJ mol^−1^ through most of the remaining conversion range. These two profiles were preserved in the leave-one-heating-rate-out test for α ≥ 0.3: Eα of neat PS decreased with conversion in every subset, and Eα of PS-NF-6 remained within 204–236 kJ mol^−1^ over the whole conversion range. The low-conversion values of neat PS are less reliable (R^2^ = 0.943 at α = 0.10) and ranged from 178 to 305 kJ mol^−1^ among the subsets.

When all four heating rates are included, PS-NF-12 shows a different profile: Eα increases from 196.8 kJ mol^−1^ at α = 0.10 to 284.0 kJ mol^−1^ at α = 0.60, while the regression quality decreases with conversion (R^2^ = 0.949 at α = 0.60 and 0.849 at α = 0.80). However, this increase is not robust. When the 5 K min^−1^ measurement is omitted, Eα of PS-NF-12 remains between 205.7 and 211.9 kJ mol^−1^ over α = 0.20–0.70 (Table 4, Figure 7d), close to the profile of PS-NF-6, whereas omitting any other heating rate preserves the increasing trend. The rising profile is therefore controlled by a single measurement. At 5 K min^−1^, the principal degradation step of PS-NF-12 was about 10 K broader (T_80_ − T_10_ = 46.1 K) than those of neat PS and PS-NF-6 (36.6 and 35.8 K), whereas at 10–20 K min^−1^ the step widths of the three samples differed by at most 7 K. This may reflect specimen-to-specimen variation in filler content or local morphology, which cannot be verified without replicate measurements. In the companion PS/Co_3_O_4_ study, which used the same PS batch and processing protocol, the 12 min composite showed a slightly decreasing Eα profile [15], consistent with the nearly constant profile obtained here when the 5 K min^−1^ measurement is omitted.

Accordingly, the KAS results do not establish that the longer mixing duration changes the conversion dependence of Eα. The increasing Eα profile of PS-NF-12 is therefore not supported by the present data set; whether it reflects a genuine kinetic feature or specimen variability at 5 K min^−1^ can only be decided by replicate measurements. The robust kinetic difference between the samples is the higher peak-region apparent activation energy of PS-NF-12 (Section 3.5).

### 3.7. Comparison with Related Systems and Limitations

Table 5 compares the present peak-based activation energies with values reported for related PS and PMMA systems. The Kissinger value of neat PS (194.3 kJ mol^−1^) is consistent with the apparent activation energy of about 200 kJ mol^−1^ reported for the main degradation region of PS under nitrogen [3] and with the Kissinger value of 182 ± 8 kJ mol^−1^ reported by Blanco et al. [19]. The increase of about 58 kJ mol^−1^ obtained for PS-NF-12 is larger than those reported for PS containing 5 wt.% POSS (11–15 kJ mol^−1^) [19] and for solution-cast PS/ZnO (−14 to +10 kJ mol^−1^, obtained with a single-heating-rate method) [20]. In contrast, the corresponding temperature shifts are modest: the characteristic temperatures of PS-NF-12 exceed those of neat PS by 5–8 K on average, whereas T_5_ increased by 31–35 K in the POSS/PS system [19] and T_50_ by up to 11 K in PS/ZnO [20]. A large change in the Kissinger activation energy combined with a small shift in peak temperature indicates that the slope is controlled by small differences in the heating-rate dependence of the peak temperatures, which is why the uncertainty analysis in Section 3.5 is essential for interpreting this value.

The comparison within the present series is also informative. In PS, the 12 min condition gave the highest peak-based activation energy both with NiFe_2_O_4_ (252.4 vs. 201.1 kJ mol^−1^ for 6 min) and with Co_3_O_4_ (231 vs. 210 kJ mol^−1^) [15], whereas in PMMA/NiFe_2_O_4_ prepared with the same protocol the 6 min condition gave the highest value (270 vs. 210 kJ mol^−1^) [14]. A plausible, but untested, explanation lies in the different degradation mechanisms of the two matrices. PMMA degrades predominantly by depolymerization initiated at chain ends and weak links, yielding mainly monomer [16]; so, its kinetics may be sensitive to local constraints near particle clusters. PS degrades by random chain scission, β-scission, and intermolecular hydrogen transfer throughout the melt [2,3,6]; so, its response may depend more on the total polymer–particle contact area and on the transport of volatile products. Because the PMMA values were obtained from DTA peaks with a different measurement protocol, this comparison is qualitative, and the hypothesis would require a matrix comparison under identical conditions.

Several mechanisms could contribute to the higher degradation temperatures and peak-region activation energy of PS-NF-12, none of which is demonstrated by the present data: (i) a larger polymer–filler contact area, if longer mixing reduced the agglomerate size, which could promote adsorption or trapping of radicals at the ferrite surface; (ii) a barrier effect that hinders the escape of volatile degradation products and becomes more pronounced as the inorganic fraction of the condensed phase increases with conversion; and (iii) the additional thermo-mechanical history of the matrix during the longer mixing. Transition-metal oxide surfaces may also catalyze degradation reactions, which would act in the opposite direction. Distinguishing among these contributions would require cross-sectional imaging with compositional mapping, molecular-weight measurements of the processed matrix, and evolved-gas analysis.

The main limitations of the study are as follows. First, one measurement was performed for each sample and heating rate; so, reproducibility was assessed only through the consistency across heating rates and the numerical tests in Section 3.5 and Section 3.6. Second, no neat PS control processed for 12 min was prepared; therefore, the effects of filler organization and of the thermo-mechanical history of the matrix cannot be separated, and the differences between PS-NF-6 and PS-NF-12 show an association with the two processing conditions rather than a causal effect of mixing duration alone. Third, the SEM observations were limited to single surface fields without compositional analysis. Fourth, the neat PS measurements were recorded several weeks before those of the nanocomposites; so, any instrumental drift between the sessions could affect the comparison with neat PS. The nanocomposites were measured within one week in an interleaved order, and their mutual comparison is less affected. Finally, only two mixing durations and one filler loading were investigated. Replicate measurements, a neat PS control for each mixing duration, and additional mixing durations are required to establish a general relationship between mixing duration and degradation kinetics. In addition, NiFe_2_O_4_ was used without surface modification. Surface functionalization of nanofillers, which has been shown to strengthen polymer–filler interactions and improve the thermal and mechanical properties of nanocomposites such as in situ prepared polyurethane/PEG-MXene systems [21], could be used in future work to separate the contributions of filler dispersion and interfacial interaction.

## 4. Conclusions

PS/NiFe_2_O_4_ nanocomposites containing 2.5 wt.% NiFe_2_O_4_ were prepared by melt blending for 6 and 12 min and compared with neat PS processed for 6 min. XRD showed that the spinel structure of NiFe_2_O_4_ was retained after processing. Surface SEM observations suggested different near-surface morphologies for the two mixing durations, but these observations were limited to single fields without compositional analysis and were not used to interpret the thermal results.

After normalization to the principal degradation step, PS-NF-12 degraded at higher temperatures than neat PS at all four heating rates and at all characteristic conversions, with mean shifts of 5–8 K, whereas the effect of PS-NF-6 was confined to the early stage of degradation. The DTG peak temperatures of the three samples differed by at most about 3 K at 10 K min^−1^.

The Kissinger apparent activation energies were 194.3 ± 18.1, 201.1 ± 14.4, and 252.4 ± 11.5 kJ mol^−1^ for neat PS, PS-NF-6, and PS-NF-12, respectively. The higher value of PS-NF-12 was preserved when any single heating rate was omitted and when the DTA peak temperatures were used instead of the DTG peak temperatures, whereas neat PS and PS-NF-6 could not be distinguished within the present uncertainty.

The KAS analysis showed a decreasing Eα profile for neat PS and a nearly constant profile for PS-NF-6, and both profiles were robust to the omission of single heating rates. The increase in Eα with conversion obtained for PS-NF-12 appeared only when the 5 K min^−1^ measurement was included; without it, Eα remained at 206–212 kJ mol^−1^ (α = 0.20–0.70), close to that of PS-NF-6. So, this increase is not supported by the present data set.

Under the investigated conditions, increasing the melt-mixing duration from 6 to 12 min was thus associated with delayed mass loss and a higher peak-region apparent activation energy of low-loading PS/NiFe_2_O_4_ nanocomposites, consistent with the behavior of PS/Co_3_O_4_ and opposite to that of PMMA/NiFe_2_O_4_ prepared with the same protocol. Because the thermal measurements were not replicated and no neat PS control processed for 12 min was available, these results show an association with the two processing conditions rather than a general relationship between mixing duration and degradation kinetics. Replicate measurements, matched neat-polymer controls, and cross-sectional compositional imaging are needed to establish the underlying mechanism.

## Figures and Tables

**Table 1:** Initial specimen mass (m_0_), characteristic temperatures from normalized TG curves, and DTG and DTA peak temperatures of neat PS and PS/NiFe_2_O_4_ nanocomposites.

Sample	β (K min^−1^)	m_0_ (mg)	T_5_ (K)	T_10_ (K)	T_30_ (K)	T_50_ (K)	T_80_ (K)	T_x_,DTG (K)	T_x_ (K)
PS	5	10.00	635.2	644.8	661.2	669.7	681.4	672.8	674.5
	10	10.66	642.3	653.4	671.2	680.2	692.5	684.6	687.2
	15	11.96	643.7	656.7	677.4	687.9	702.1	695.2	697.8
	20	14.22	655.2	665.5	683.1	692.8	706.8	697.6	703.0
PS-NF-6	5	16.38	636.6	646.1	661.1	669.6	681.9	671.6	674.3
	10	14.75	647.1	657.1	673.2	681.5	694.0	684.2	687.0
	15	14.22	650.5	661.6	678.9	687.4	699.8	689.8	695.5
	20	15.41	657.7	667.8	684.6	693.5	707.0	697.7	703.1
PS-NF-12	5	10.37	637.6	648.9	668.0	678.1	695.0	678.5	681.3
	10	14.60	649.6	660.5	676.1	684.3	697.2	687.6	690.1
	15	13.27	658.7	668.7	683.9	692.8	707.8	694.9	699.3
	20	16.41	662.2	672.7	688.1	696.1	709.4	698.9	704.4

T_5_–T_80_ are the temperatures at α = 0.05–0.80, with α defined by Equation (1); T_x_,DTG and T_x_ are the DTG and DTA peak temperatures, respectively.

**Table 2:** Peak-based apparent activation energies of neat PS and PS/NiFe_2_O_4_ nanocomposites.

Sample	Kissinger E_a_ (kJ mol^−1^)	R^2^	Takhor E_a_ (kJ mol^−1^)	R^2^	Kissinger E_a_, DTA Peaks (kJ mol^−1^)	R^2^
PS	194.3 ± 18.1	0.983	205.7 ± 18.1	0.985	176.1 ± 8.7	0.995
PS-NF-6	201.1 ± 14.4	0.990	212.4 ± 14.4	0.991	180.0 ± 8.6	0.995
PS-NF-12	252.4 ± 11.5	0.996	263.8 ± 11.5	0.996	221.4 ± 20.2	0.984

Values are given as slope ± standard error of the linear regression (*n* = 4). The first two methods use the DTG peak temperatures (T_x_,DTG).

**Table 3:** Sensitivity of the Kissinger apparent activation energy (kJ mol^−1^) to the omission of single heating rates and to perturbation of the DTG peak temperatures.

Sample	All Four Rates	5 K min^−1^ Omitted	10 K min^−1^ Omitted	15 K min^−1^ Omitted	20 K min^−1^ Omitted	95% Interval (σ = 1 K)
PS	194.3	182.1	196.1	207.1	180.5	174–215
PS-NF-6	201.1	189.6	200.6	195.9	217.5	180–225
PS-NF-12	252.4	231.0	255.0	256.2	252.0	221–289

**Table 4:** KAS apparent activation energies (Eα, kJ mol^−1^) and coefficients of determination. The last two columns give PS-NF-12 with the 5 K min^−1^ measurement omitted.

α	PS Eα	R^2^	PS-NF-6 Eα	R^2^	PS-NF-12 Eα	R^2^	PS-NF-12 (5 K min^−1^ Omitted) Eα	R^2^
0.10	232.7	0.943	223.5	0.991	196.8	0.997	194.0	0.989
0.20	236.8	0.986	217.5	0.996	225.9	0.997	208.7	0.994
0.30	229.4	0.997	213.4	0.998	246.5	0.988	209.5	0.992
0.40	222.7	0.999	214.7	0.998	261.4	0.979	211.3	0.987
0.50	217.9	0.998	215.5	0.997	272.9	0.967	211.9	0.975
0.60	213.2	0.997	216.6	0.996	284.0	0.949	210.9	0.958
0.70	208.9	0.995	217.1	0.995	296.5	0.913	205.7	0.938
0.80	202.1	0.994	214.9	0.994	299.5	0.849	193.2	0.900

**Table 5:** Peak-based apparent activation energies of the present samples compared with related systems.

System	Preparation	Filler Content	Method	E_a_, Matrix (kJ mol^−1^)	E_a_, Composite(s) (kJ mol^−1^)	Ref.
PS/NiFe_2_O_4_	Melt blending, 6/12 min	2.5 wt.%	Kissinger, DTG peaks	194.3	201.1/252.4	This work
PS/NiFe_2_O_4_	Melt blending, 6/12 min	2.5 wt.%	Kissinger, DTA peaks	176.1	180.0/221.4	This work
PS/Co_3_O_4_	Melt blending, 6/12 min	2.5 wt.%	Kissinger	194	210/231	[15]
PMMA/NiFe_2_O_4_	Melt blending, 6/12 min	2.5 wt.%	Kissinger, DTA peaks	189	270/210	[14]
PS/POSS	In situ polymerization	5 wt.%	Kissinger, DTG peaks, N_2_	182	193, 197	[19]
PS/ZnO	Solution casting	0.5–3.0 wt.%	Coats–Redfern, 10 K min^−1^	263.0	248.8–272.6	[20]

## Data Availability

The original contributions presented in this study are included in the article. Further inquiries can be directed to the corresponding author.

## Abbreviations

The following abbreviations are used in this manuscript:

Symbol/Abbreviation	Meaning
PS	Polystyrene
PS-NF-6	PS/NiFe_2_O_4_ nanocomposite containing 2.5 wt.% NiFe_2_O_4_ and melt-mixed for 6 min
PS-NF-12	PS/NiFe_2_O_4_ nanocomposite containing 2.5 wt.% NiFe_2_O_4_ and melt-mixed for 12 min
XRD	X-ray diffraction
SEM	Scanning electron microscopy
DTA	Differential thermal analysis
TG	Thermogravimetry
DTG	Derivative thermogravimetry
KAS	Kissinger–Akahira–Sunose
Mw	Weight-average molecular weight
α	Degree of conversion
β	Heating rate
Ea	Apparent activation energy
Eα	Conversion-dependent apparent activation energy
T_5_, T_10_, T_30_, T_50_, T_80_	Temperatures at α = 0.05, 0.10, 0.30, 0.50, and 0.80 (Equation (1))
Tα	Temperature corresponding to a given degree of conversion, α
T_x_	DTA degradation-peak temperature
T_x_,DTG	Temperature of the maximum mass-loss rate (DTG peak)
